# Supplementary material for: Sequence anticipation and spike-timing-dependent plasticity emerge from a predictive learning rule
Source: Nat Commun. 2023 Aug 21;14:4985. doi: 10.1038/s41467-023-40651-w (PMC10442404; doi:10.1038/s41467-023-40651-w)
Supplement: Supplementary file 1 — Supplementary Information [file 41467_2023_40651_MOESM1_ESM.pdf]

Supplementary Information for: "Sequence anticipation and spike-timing-dependent plasticity emerge from a predictive learning rule"

Matteo Saponati[a,b,c,d], Martin Vinck[a,c,d]

[a]*Ernst-Strüngmann Institute for Neuroscience in Cooperation with Max Planck Society, Frankfurt Am Main, Germany*
[b]*IMPRS for Neural Circuits, Max-Planck Institute for Brain Research, Frankfurt Am Main, Germany*
[c]*Donders Centre for Neuroscience, Department of Neuroinformatics, Radboud University Nijmegen, 6525 Nijmegen, the Netherlands*
[d]*Correspondence: matteo.saponati@esi-frankfurt.de, martin.vinck@esi-frankfurt.de*

Contents

1 Supplementary Figures **1**

2 Supplementary Notes **12**
2.1 Relations with STDP models 12
2.2 Relations with STDP experiments 12

3 Supplementary References **14**

1. Supplementary Figures

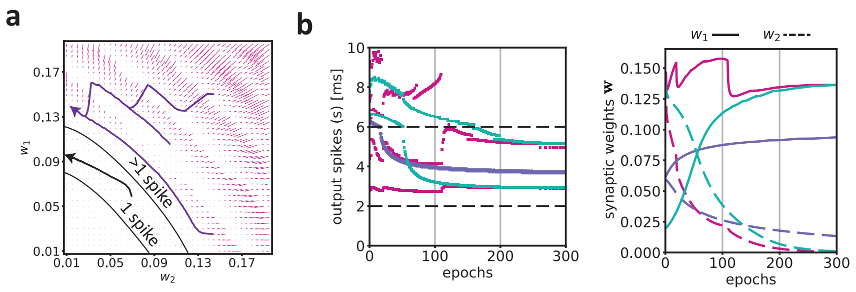

Figure S1: Dynamics in the parameter space. Relates to Figure 1 of the main text. a) We show here that the learning dynamics of the model shown in Figure 1c-d are qualitatively the same when the initial conditions lie in regions of multiple output spikes. Shown is the flow field as in Figure 1d. The black arrow shows the trajectory of the weights obtained by training the model with four different initial conditions, in particular $\mathbf{w}_0 = (0.06, 0.06)$, $\mathbf{w}_0 = (0.1, 0.1)$, $\mathbf{w}_0 = (0.15, 0.02)$, $\mathbf{w}_0 = (0.15, 0.15)$. It can be seen, that all the regions of the parameter space with two or more post-synaptic spikes converge to a fixed point, at which there is maximal credit for the first input (i.e. $w_1 > w_2$). **b**) Right: same as in Figure 1c, for the four initial conditions shown in **a**.

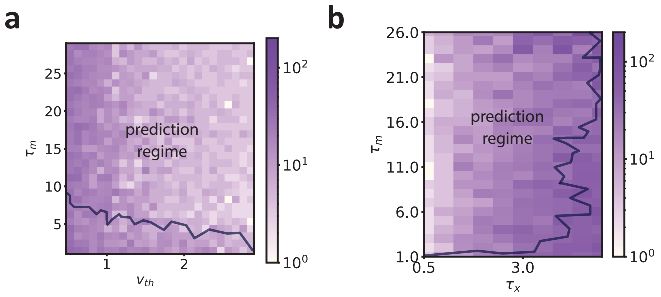

Figure S2: Effect of model parameters. Relates to Figure 2 of the main text. **a**) Simulations were performed as in Figure 2, but now with different values of model parameters τ_m and v_{th}. The color map corresponds to the total number of output spikes at the end of learning. We show regions for the parameter space where the total number of spikes was smaller than 200. The black line outlines the region of parameter space where the neuron (in epoch 1000) fires between 2 and 10 ms after the onset of the input sequence, which can be interpreted as the predictive or anticipatory solution. For the vast portion of the parameter space, the model converges to the anticipatory solution. **b**) Same as in **a** for different values of model parameters τ_m and τ_x.

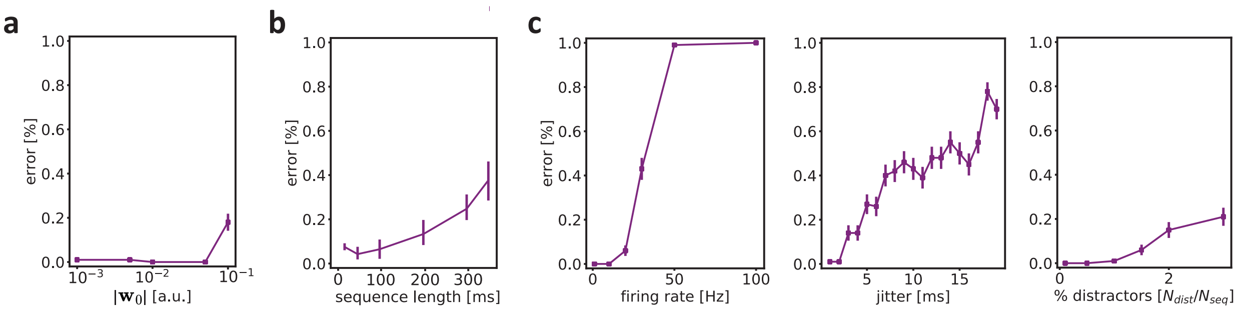

Figure S3: Dependence of performance, in terms of anticipation of sequences, depending on model parameters. Relates to Figure 2 of the main text. **a**) Simulations were performed as in Figure 2. To quantify the performance in terms of anticipating the sequence, we labeled successful simulation based on the criteria of input selectivity and fast anticipation (see Methods). Performance is then shown as the fraction of simulations in which the model did not reach an anticipatory solution. Performance is shown as a function of the initial weight vector $\mathbf{w}_0$, with the weight the same for each pre-synaptic input. The performance does not strongly depend on the initial conditions. For each weight, we performed 100 simulations (shown are means and the standard errors of the mean). For each simulation, we used 2000 epochs. **b**) As **a**, but now as a function of the sequence length. For each sequence length, we performed 100 simulations (shown are means and the standard errors of the mean). **c**) Left: Performance as a function of the maximal rate of background firing. Center: Performance as a function of the maximal spike time jitter. Right: Performance as a function of the proportion of pre-synaptic distractor neurons, that is pre-synaptic neurons which do not participate in the sequence. For each value of firing rate, jitter and % of distractors, we performed 100 simulations (shown are means and the standard errors of the mean).

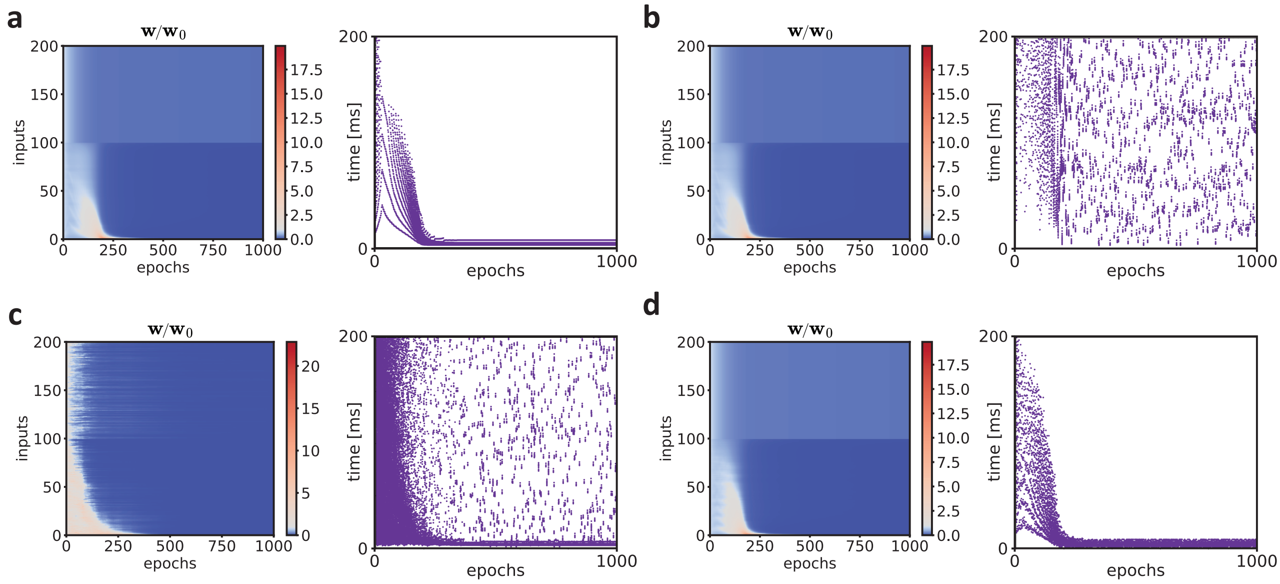

Figure S4: Effect of individual noise sources - Relates to Figure 2 of the main text. **a**) Simulations were performed as in Figure 2, however without any noise source (no spike time jitter, no distractor neurons, no background firing, no random onset of the sequence). Dynamics of the synaptic weights **w** (left) and of the post-synaptic spiking activity (right) across training, without any source of noise. **b**) Same as in **a**, but now with one noise source, in this case the sequence onset drawn from a uniform distribution with values between 0 and 100 ms. **c**) Same as in **a**, with now one noise source, namely random background firing following an homogeneous Poisson process with rate distributed between 0 and 10 Hz. **d**) Same as in **a**, with again one noise source, namely jitter of the input spike times (random jitter between -2 and 2 ms).

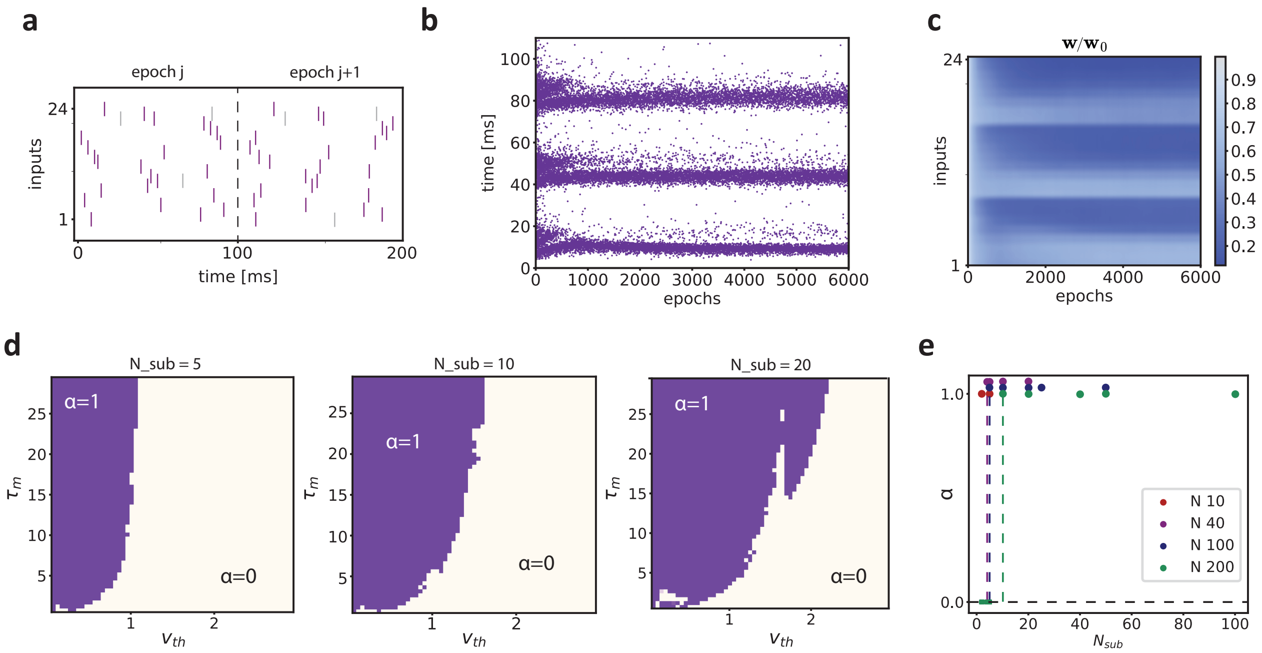

Figure S5: Learning multiple independent sequences and the capacity of the model. Relates to Figure 2 of the main text. **a**) Example spike sequence during different training epochs. The input spike trains contain a sequence given by the correlated activity of 24 pre-synaptic neurons that firex sequentially with relative delays of 2 ms (purple spike pattern). Each epoch contains two different sources of noise: (1) jitter of the spike times (random jitter between -2 and 2 ms, which is applied randomly in each epoch); and (2) random firing following an homogeneous Poisson process with rate $\lambda = 5$ Hz. The total sequence is divided into 3 sub-sequences of $N = 8$ separated by 20 ms. **b**) Dynamics of the post-synaptic spiking activity during learning for the four different sub-sequences. The neuron learns to represent several components of the input structure by potentiating the unpredictable part, i.e. the start, of each sub-sequence. **c**) Dynamics of the synaptic weights during training. The synaptic weights are ordered from 1 to 24 following the temporal order of the sequence. The synaptic weights corresponding to the first inputs of each sub-sequence are maximally potentiated. **d**) Capacity of the model α for different values of model parameters τ_m (membrane time constant) and v_{th} (spiking threshold). The capacity α is defined as the percentage of sub-sequences that the neuron anticipates at the end of training (same performance criterion as in Figure S3). The sequence was composed by the subsequent firing of $N = 100$ pre-synaptic inputs, divided in N_{sub} sub-sequences with $N_{sub} = 5$, $N_{sub} = 10$ and $N_{sub} = 20$ from left to right. In each condition, the neuron anticipates every sub-sequence in the input for a broad range of model parameters. **e**) Capacity of the model as a function of the number of sub-sequences N_{sub} for a different total number of pre-synaptic neurons N. Here we fixed $v_{th} = 1$ and $\tau_m = 20$ ms. Each color corresponds to a different total number of pre-synaptic neurons. We then quantified the capacity for different lengths of the sub-sequences (i.e. N_{seq}). For example, there are 2 sub-sequences of length N_{seq} for a total number of 200 pre-synaptic neurons. The figure shows that full capacity (i.e. $\alpha = 1$) can be reached even for small sub-sequences, and for both small and large numbers of pre-synaptic neurons. Values higher than $\alpha = 1$ correspond to values of $\alpha = 1$ and are shown as such for visualization purposes.

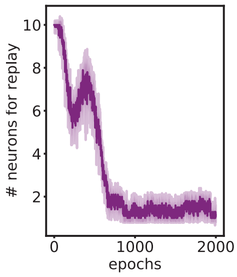

Figure S6: Number of neurons in the network that need to be activated for sequence recall to occur. Relates to Figure 3 of the main text. The y-axis corresponds to the number of neurons in the network that need to be activated by the input sequence in order for recall to occur. For each epoch, we tested how many neurons needed to be activated by the corresponding pre-synaptic sequence to obtain full recall. First, we presented the pre-synaptic sequence corresponding to the first neuron in the network. We observed how many neurons in the network were active after the sequence presentation. Note that if a neuron is not activated by the pre-synaptic sequence, the pre-synaptic neurons still exhibit background firing. Then, we systematically increased the number of neurons in the network which received the corresponding pre-synaptic sequence. We examined the minimum number of neurons in the network that needed to be active such that every other neuron in the network also fired sequentially. We repeated this analysis for each training epoch and for 100 simulations with different noise realizations. At the beginning of training, each sequence input to each neuron in the network is needed to obtain a full recall of the sequence. At the end of training, only the inputs to the first neuron are required to trigger a full recall of the input sequence in the network.

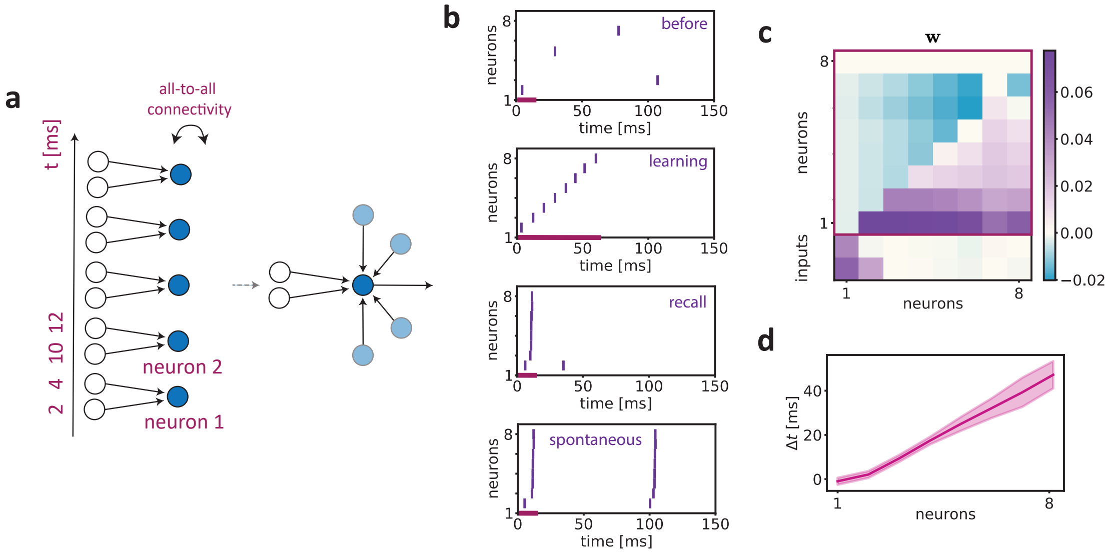

Figure S7: Anticipation and recall of sequences with all-to-all connected network. Relates to Figure 3 of the main text. We explored the dynamics of a network model where $N = 8$ neurons received an input sequence distributed across a total of 16 pre-synaptic neurons. Each neuron in the network received 2 pre-synaptic inputs The pre-synaptic neurons fired in a sequential manner with delays of 2 ms. Each epoch contains two different sources of noise: (1) jitter of the spike times (random jitter between -2 and 2 ms, which is applied randomly in each epoch); and (2) random firing following an homogeneous Poisson process with rate $\lambda = 10$ Hz. The network had a recurrent, all-to-all connectivity scheme, and is arranged along the time dimension of the sequence, such that each neuron receives part of the input in a subsequent manner. The sequence onset of pre-synaptic inputs for the $n+1$-th neuron started 8 ms after the sequence onset for the n-th neuron in the network, etc. Accordingly, the pattern that each neuron tries to predict is composed of the external pre-synaptic input and the internally generated activity of the network. **a**) Illustration of the network model, where only 5 neurons of the network are represented for simplicity. **b**) Top: Network spiking activity when presenting only part of the input sequence, namely the sequential input to the first 3 neurons in the network. Note that the other network neurons still received inputs from their corresponding pre-synaptic neurons due to stochastic background firing. Middle: Network spiking activity during the first training epoch, when the entire sequence was presented. Bottom: The first plot shows the network spiking activity at the end of training (epoch 2000) when we presented only part of the sequence (as in the top plot). This figure shows that after learning, the network learns to recall the entire sequence. The second plot shows the network spiking activity when we presented only part of the sequence, but also the end of the sequence (i.e. activating the last two network neurons). There is no effect of activating the neurons at the end of the sequence. Note that random background firing can also elicit spontaneous recall of the sequence, outside the stimulation period. **c**) The synaptic weight matrix obtained at the end of training (epoch 1000). The i-th column (and the top 10 rows) corresponds to the synaptic weights learned from the other neurons to the i-th neuron in the network. The bottom 2 entries correspond to the weights for the pre-synaptic inputs. **d**) Difference between the latency of the first spike of each single neuron in the first epoch of training and after training. Positive values indicate that an earlier latency due to training. The panel shows the mean and standard deviation computed over 100 different simulations.

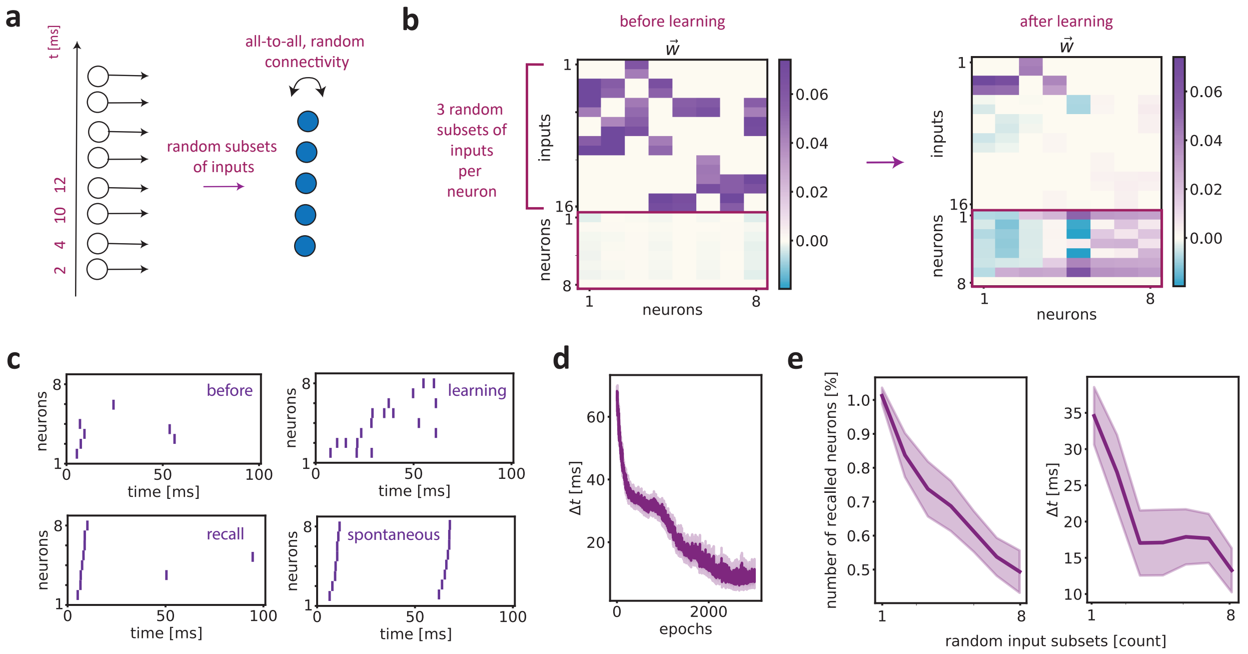

Figure S8: Anticipation and recall of sequences with randomly connected network. Relates to Figure 3 of the main text. We explored the dynamics of a network model where $N = 8$ neurons received an input sequence distributed across a total of 16 pre-synaptic neurons. The pre-synaptic neurons are divided in 8 subsets of 2 pre-synaptic neurons that fired sequentially with a relative delay of 2 ms. The sequence onset of pre-synaptic inputs for the $j + 1$-th subset started 8 ms after the sequence onset of the j-th subset. For example, the pre-synaptic neurons of the first subset fired at 2 and 4 ms, the pre-synaptic neurons of the second subset fired at 10 and 12 ms, etc. Each epoch contains two different sources of noise: (1) jitter of the spike times (random jitter between -2 and 2 ms, which is applied randomly in each epoch); and (2) random firing following an homogeneous Poisson process with rate $\lambda = 5$ Hz. Each neuron in the network received randomly chosen input subsets. The network had a recurrent, all-to-all connectivity scheme, and the recurrent connections were initialized at random following a homogeneous distribution between 0 and a maximal value. Accordingly, the pattern that each neuron tries to predict is composed of the random subsets of pre-synaptic inputs and the internally generated activity of the network. **a**) Illustration of the network model, where only 5 neurons of the network are represented for simplicity. **b**) The synaptic weight matrix before learning and at the end of learning (epoch 3000). The bottom 8 entries of the i-th column correspond to the synaptic weights from the other neurons to the i-th neuron in the network. The top 16 entries correspond to the weights for the pre-synaptic inputs to the i-th neuron in the network, where only the inputs from the randomly chosen subsets had non-zero values. **c**) Raster plot of the network's activity during different epochs of training: (1) The "before" case, where only the pre-synaptic neurons corresponding to the first two input subsets exhibited sequential firing. (2) The "learning" or conditioning case, where we presented the entire sequence (which was repeated 3000 times). (3) The "after" or "recall" condition, which was the same as the before condition (now after learning). (4) Same as (3), but an example where spontaneous recall occurs due to the background stochastic firing. The neurons are ordered following their firing times. **d**) Evolution of the duration of network activity across epochs. We computed the temporal difference between the last spike of the last neuron and the first spike of the first neuron to estimate the total duration of the network's activity. The panel shows the mean and standard deviation computed over 100 different simulations. **e**) Left: percentage of neurons in the network that were active after learning (epoch 3000), as a function of the number of random subsets of inputs to each neuron in the network. Here, only the pre-synaptic neurons corresponding to the first two input subsets exhibited sequential firing, as in the "before" case in panel **c**. Right: duration of the network activity as a function of the number of random subsets of inputs to each neuron in the network. We computed the duration of the network activity as in panel **d**.

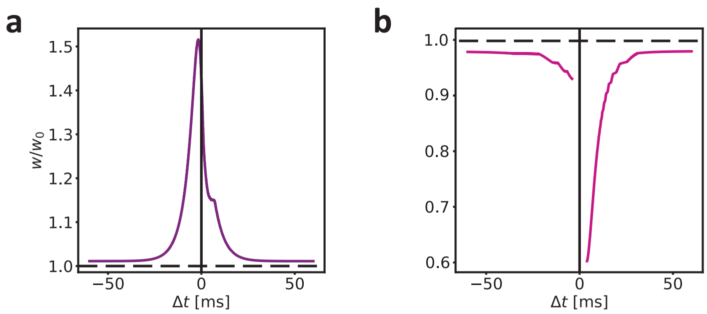

Figure S9: Different STDP kernels. Relates to Figure 4 of the main text. **a**) Weight change (in percentage) of the sub-threshold input as a function of the delay between the two input spikes (see Methods). We obtained the learning window by setting $\tau = 5$ ms, $v_{th} = 0.4$ and initial conditions $w_1 = 0.0007$ and $w_2 = 0.02$. **b**) Weight change (in percentage) of the sub-threshold input as a function of the delay between the two input spikes (see Methods). We obtained the learning window by setting $\tau = 10$ ms, $v_{th} = 2$ and initial conditions $w_1 = 0.02$ and $w_2 = 0.1$.

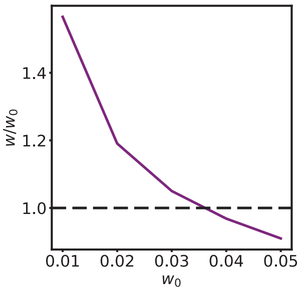

Figure S10: Dependence of STDP on the initial synaptic weight. Relates to Figure 4 of the main text. Simulations were performed as in Figure 4b, however we simulated different initial conditions for the synaptic weight corresponding to the sub-threshold input. Weight change (in percentage) as a function of different initial values for the synaptic weights.

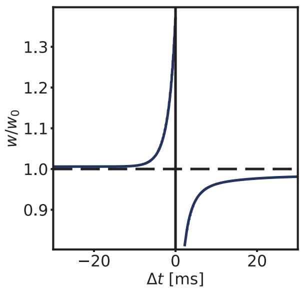

Figure S11: Spike-timing-dependent-plasticity with fixed synapses. Relates to Figure 4 of the main text. Simulations were performed as in Figure 3, however, we fixed the synaptic weight of the supra-threshold input, that is the one eliciting a spike. Only the weight of the sub-threshold input was plastic. Weight change (in percentage) as a function of the delay between the two input spikes.

2. Supplementary Notes

2.1. Relations with STDP models

We further discuss what are the relations between the predictive plasticity rule described in the main text and phenomenological models for STDP. In particular, we focus on what STDP models can and cannot reproduce in terms of the results presented in the main text.

1. STDP rules lead to unlimited increase or decrease of synaptic weights, and additional stability mechanisms are typically required [1–3]. For example, it is common to artificially limit the set of possible weight values or to include homeostatic plasticity rules [3, 4]. However, stability mechanisms can abolish the competitive interaction between synapses that is usually at the basis of STDP phenomena [4–6], such as the decrease in output latency [7–9]. A specific combination of STDP rules and regulatory mechanisms is usually required to obtain specific results. For example, for the decrease in the response latency for incoming input spike trains in [8, 9], the authors used an STDP rule with a specific learning window, and with internal regulatory mechanisms: (1) the authors used the Restricted Nearest-Neighbors (Restricted-NN) STDP rule, where a weight change occurs only for the first post-synaptic spike immediately following or preceding a pre-synaptic spike - that is, potentiation and depression alternates in time - and (2) the authors used a specific STDP window which is biased towards de-potentiation. These regulatory/homeostatic mechanisms are necessary to reproduce the results in [10]. To the best of our knowledge, [8, 9] are the only works showing anticipation of spike sequences similar to the results presented in the main text.
2. Experimental evidence has shown that higher-order spike patterns (beyond pre-post pairing) during STDP have diverse effects [11–14]. STDP rules with restricted spike-spike interactions only allow weight updates for a limited number of coincidences between pre- and post-synaptic spikes. As a result, these STDP rules are unable to replicate how plasticity behaves with higher-order spike patterns or how plasticity is influenced by the frequency of pre-post pairings [15]. In contrast, the predictive plasticity rule proposed in this manuscript is capable of reproducing both of these effects. Only unrestricted STDP rules that explicitly consider higher-order spike interactions [16] or explicitly depend on the post-synaptic membrane voltage [17] can also account for higher-order STDP effects.
3. The predictive learning rule described in the main text is capable of generating different types of STDP kernels, such as asymmetrical or symmetrical. This cannot be possible if one would consider STDP-like learning rules, as they are defined by specific learning windows [5, 8, 9].
4. The predictive learning rule is determined by the gradient of an objective function, leading to weight updates that converge to specific fixed points regardless of initial conditions. By that means, our model replicates the experimental observation that the amount of Long-Term Potentiation (LTP) depends on the initial strength of the synaptic input, a phenomenon observed experimentally [11, 18], and is not reproducible with STDP rules that do not directly depend on synaptic weights [5].

2.2. Relations with STDP experiments

We further discuss the link between the predictive learning rule described in the main text and STDP experiments in both *in-vivo* and *in-vitro*.

1. Experimental evidence suggests that STDP is more broadly defined by changes in the post-synaptic membrane potential that occur at different moments in time [19–24] and that in turn can trigger different molecular cascades [25–28]. In STDP protocols *in-vitro*, synaptic plasticity depends on the timing between voltage changes due to pre-synaptic inputs, and voltage changes due to current injection and associated back-propagating action potential (bAP). The proposed learning rule depends on the temporal relations between voltage changes caused by different pre-synaptic inputs, and other sources of voltage changes - such as current injection, or bAPs - can also influence plasticity. By its very nature, the predictive learning rule can thereby reproduce STDP mechanisms in line with experimental evidence *in-vitro*, as well as give rise to timing-dependent phenomena, such as sequence anticipation and sequence recall at compressed timescales, that might take place *in-vivo* [29, 30].
2. The predictive plasticity rule does reproduce several experimental observations of STDP, both qualitatively and quantitatively. Accordingly, the phenomenological model that can, to our knowledge, reproduce the majority of experimental observations, indeed involves the membrane potential as a pivotal variable for STDP (the work from Clopath et al. [17]) (see also the Discussion section in the main text).

3. The predictive learning rule is not independent of post-synaptic spikes. On the contrary, it crucially depends on the temporal relationships between inputs, where LTP or LTD is observed depending on the timing of the post-synaptic spikes. Every time a spike is emitted, the membrane potential receives negative feedback, mimicking the spiking reset mechanism. This reset mechanism crucially influences learning, and it is one of the bases of the results in the main text.

3. Supplementary References

References

1. Oja, E. Simplified neuron model as a principal component analyzer. *Journal of mathematical biology* **15,** 267–273 (1982).
2. Abbott, L. F. & Nelson, S. B. Synaptic plasticity: taming the beast. *Nature neuroscience* **3,** 1178–1183 (2000).
3. Van Rossum, M. C., Bi, G. Q. & Turrigiano, G. G. Stable Hebbian learning from spike timing-dependent plasticity. *Journal of neuroscience* **20,** 8812–8821 (2000).
4. Miller, K. D. & MacKay, D. J. The role of constraints in Hebbian learning. *Neural computation* **6,** 100–126 (1994).
5. Morrison, A., Diesmann, M. & Gerstner, W. Phenomenological models of synaptic plasticity based on spike timing. *Biological cybernetics* **98,** 459–478 (2008).
6. Chistiakova, M., Bannon, N. M., Bazhenov, M. & Volgushev, M. Heterosynaptic plasticity: multiple mechanisms and multiple roles. *The Neuroscientist* **20,** 483–498 (2014).
7. Song, S., Miller, K. D. & Abbott, L. F. Competitive Hebbian learning through spike-timing-dependent synaptic plasticity. *Nature neuroscience* **3,** 919–926 (2000).
8. Guyonneau, R., VanRullen, R. & Thorpe, S. J. Neurons tune to the earliest spikes through STDP. *Neural Computation* **17,** 859–879 (2005).
9. Masquelier, T., Guyonneau, R. & Thorpe, S. J. Spike timing dependent plasticity finds the start of repeating patterns in continuous spike trains. *PloS one* **3,** e1377 (2008).
10. Hathway, P. & Goodman, D. [Re] Spike Timing Dependent Plasticity Finds the Start of Repeating Patterns in Continuous Spike Trains. *ReScience* **4.** http://dx.doi.org/10.5281/zenodo.1327348 (2018).
11. Sjöström, P. J., Turrigiano, G. G. & Nelson, S. B. Rate, timing, and cooperativity jointly determine cortical synaptic plasticity. *Neuron* **32,** 1149–1164 (2001).
12. Froemke, R. C. & Dan, Y. Spike-timing-dependent synaptic modification induced by natural spike trains. *Nature* **416,** 433–438 (2002).
13. Froemke, R. C., Tsay, I. A., Raad, M., Long, J. D. & Dan, Y. Contribution of individual spikes in burst-induced long-term synaptic modification. *Journal of neurophysiology* (2006).
14. Feldman, D. E. The spike-timing dependence of plasticity. *Neuron* **75,** 556–571 (2012).
15. Nevian, T. & Sakmann, B. Spine Ca2+ signaling in spike-timing-dependent plasticity. *Journal of Neuroscience* **26,** 11001–11013 (2006).
16. Pfister, J.-P. & Gerstner, W. Triplets of spikes in a model of spike timing-dependent plasticity. *Journal of Neuroscience* **26,** 9673–9682 (2006).
17. Clopath, C., Büsing, L., Vasilaki, E. & Gerstner, W. Connectivity reflects coding: a model of voltage-based STDP with homeostasis. *Nature neuroscience* **13,** 344 (2010).
18. Bi, G.-q. & Poo, M.-m. Synaptic modifications in cultured hippocampal neurons: dependence on spike timing, synaptic strength, and postsynaptic cell type. *Journal of neuroscience* **18,** 10464–10472 (1998).
19. Scanziani, M., Malenka, R. C. & Nicoll, R. A. Role of intercellular interactions in heterosynaptic long-term depression. *Nature* **380,** 446–450 (1996).
20. Artola, A. & Singer, W. Long-term depression of excitatory synaptic transmission and its relationship to long-term potentiation. *Trends in neurosciences* **16,** 480–487 (1993).
21. Golding, N. L., Staff, N. P. & Spruston, N. Dendritic spikes as a mechanism for cooperative long-term potentiation. *Nature* **418,** 326–331 (2002).
22. Royer, S. & Paré, D. Conservation of total synaptic weight through balanced synaptic depression and potentiation. *Nature* **422,** 518–522 (2003).

23. Harvey, C. D., Yasuda, R., Zhong, H. & Svoboda, K. The spread of Ras activity triggered by activation of a single dendritic spine. *Science* **321,** 136–140 (2008).

24. Lisman, J. & Spruston, N. Questions about STDP as a general model of synaptic plasticity. *Frontiers in synaptic neuroscience* **2,** 140 (2010).

25. Lisman, J. A mechanism for the Hebb and the anti-Hebb processes underlying learning and memory. *Proceedings of the National Academy of Sciences* **86,** 9574–9578 (1989).

26. Yang, S.-N., Tang, Y.-G. & Zucker, R. S. Selective induction of LTP and LTD by postsynaptic [Ca2+] i elevation. *Journal of neurophysiology* **81,** 781–787 (1999).

27. Lisman, J., Yasuda, R. & Raghavachari, S. Mechanisms of CaMKII action in long-term potentiation. *Nature reviews neuroscience* **13,** 169–182 (2012).

28. Graupner, M. & Brunel, N. Calcium-based plasticity model explains sensitivity of synaptic changes to spike pattern, rate, and dendritic location. *Proceedings of the National Academy of Sciences* **109,** 3991–3996 (2012).

29. Diba, K. & Buzsáki, G. Forward and reverse hippocampal place-cell sequences during ripples. *Nature neuroscience* **10,** 1241–1242 (2007).

30. Gavornik, J. P. & Bear, M. F. Learned spatiotemporal sequence recognition and prediction in primary visual cortex. *Nature neuroscience* **17,** 732–737 (2014).
